# Hyperammonemia: A Report of Maternal Biliary Cirrhosis and Neonatal Outcome

**DOI:** 10.1155/2013/507169

**Published:** 2013-02-12

**Authors:** Deana J. Hussamy, David B. Nelson, Stephan A. Shivvers

**Affiliations:** Department of Obstetrics and Gynecology, University of Texas Southwestern Medical Center, Parkland Health and Hospital System, 5323 Harry Hines Boulevard, Dallas, TX 75390-9032, USA

## Abstract

Although uncommon during pregnancy, cirrhosis results in multiple medical complications impacting both mother and fetus. Previous reports suggest liver dysfunction in pregnancy causes accumulation of neurotoxins within the maternal compartment that increases neonatal morbidity through placental transfer. We present a case of a 36-year-old G_2_P_1_ female with history of biliary cirrhosis presenting at 32-weeks' gestation with hepatic congestion progressing to hepatic encephalopathy prompting delivery. Umbilical cord sampling and postnatal infant testing demonstrated elevated ammonia levels which resolved by 12 hours of life without intervention. At discharge, the infant did not demonstrate evidence of neurologic deficit. We conclude that acute maternal hepatic encephalopathy and hyperammonemia due to chronic liver disease do not portend adverse neonatal outcomes, notably encephalopathy.

## 1. Introduction

 Due to deleterious effects of maternal hepatic derangements on fertility, pregnancy is uncommon in women with advanced liver dysfunction [1]. When pregnancy does occur, poor maternal and infant outcomes have been reported [1–3]. Additionally, infant complications related to metabolic derangements, such as inherited uric acid cycle dysfunction, have been described to cause hyperammonemia leading to encephalopathy [4]. We now present a case of pregnancy complicated by iatrogenic biliary cirrhosis with worsening hepatic congestion that resulted in delivery of a viable infant without neurological consequences from in utero exposure of chronic maternal hyperbilirubinemia and acute hyperammonemia. 

## 2. Case

A 36-year-old, gravida 2, para 1, Hispanic female at 11-weeks' gestation presented with a twelve-year history of iatrogenic biliary cirrhosis following a common bile duct injury during a previous cholecystectomy. Persistent hepatobiliary dysfunction necessitated transjugular intrahepatic portosystemic shunt (TIPS) for esophageal varices and percutaneous biliary drainage with radiographically guided exchange of biliary drainage tubes every three months. She denied prior episodes of hepatic encephalopathy or neurologic deficiencies and was using a titrated lactulose therapy. Due to these co-morbidities, she was referred to our high-risk, Maternal-Fetal Medicine service for continued antepartum care with additional multidisciplinary support from Cardiology, Hepatology, Interventional Radiology (IR), and Pain/Palliative Care. Concerns related to radiologic risks to pregnancy led IR to defer further biliary drain exchange until completion of pregnancy. 

 At 32-weeks' gestation, she presented with shortness of breath, midepigastric pain, and persistent nausea/vomiting. On exam, she was afebrile and alert with mild jaundice, scleral icterus, functioning biliary drains, and lower-extremity petechiae. Laboratory studies are shown in Table 1. EKG, chest, and abdominal plain films were unremarkable. Initially, her symptomatology was attributed to hypervolemia and hepatic congestion without overt decompensation. She was diuresed with prompt response but remained hospitalized for further observation. During admission, her biliary drains were noted to be malfunctioning with concern for obstruction. Despite elevated ammonia levels (82–125 mcmol/L), the patient remained alert. The drains were replaced with clinical improvement. Fetal status remained overall reassuring with corticosteroids administered due to intermittent threats of preterm labor. 

On hospital day 12, at 33^5/7^-weeks' gestation, the patient became obtunded with evidence of hepatic encephalopathy and marked hyperammonemia (314 mcmol/L) that necessitated emergent intubation and transfer to the Medical Intensive Care Unit. Head CT, EKG, and CXR were performed without acute cardiopulmonary or neurological findings. Empiric antibiotics were initiated for presumptive cholangitis and biliary sepsis, with lactulose administered aggressively for management of hyperammonemia. A multidisciplinary discussion was conducted with the patient's family regarding rapid maternal decompensation with a decision to expedite delivery. On hospital day 13, at 33^6/7^-weeks' gestation, a cesarean delivery of a 1977-gram female infant was performed. Golden-colored amniotic fluid was noted at delivery. Uterine atony required several doses of uterotonics, as well as transfusion of one unit of packed red blood cells, six units of platelets, and two units of fresh-frozen plasma. Following delivery, maternal mental status rapidly improved with decline in serum ammonia levels (53 mcmol/L). She was extubated and discharged home in stable condition by postoperative day 5. 

Following delivery, the infant was transferred to the Neonatal Intensive Care Unit for respiratory support and observation. Umbilical cord levels of ammonia were elevated (79 mcmol/L) but rapidly improved (46 mcmol/L) by 7 hours of life without intervention. Infant hospitalization was prolonged due to respiratory complications of prematurity and pneumonia. Throughout hospitalization, there was no evidence of neurological deficit, and the infant was discharged home in stable condition on day of life 37. 

## 3. Comment

 Historically, poor fecundity in women with chronic liver disease has limited available data in pregnancy [3]. Compounded with the disruption of the hypothalamic-pituitary axis, cirrhosis typically affects women well beyond childbearing age, which further explains the rarity of this condition [5]. As care improves, the number of pregnancies complicated by cirrhosis is likely to increase [3]. We present this case to add to the paucity of data regarding management strategies and maternal-infant outcomes from in utero exposure to maternal hyperbilirubinemia and hyperammonemia.

 Previous reports describe maternal hyperbilirubinemia and neonatal risks of kernicterus as related to neurotoxicity from unconjugated bilirubin passing the blood-brain barrier [6]. Although animal models have described free passage of unconjugated bilirubin across the placenta, a preferential diffusion gradient towards the maternal compartment establishes a maternal role in fetal bilirubin elimination [7]. With maternal liver dysfunction, this balance is disrupted and fetal bilirubin elimination is impaired [8]. Cotton and colleagues reported a case of cirrhosis complicating pregnancy leading to fulminant liver failure with hepatic encephalopathy; delivery resulted in an infant without evidence of kernicterus. The authors suggested the extent of maternal hepatic dysfunction necessary to elevate unconjugated bilirubin levels to cause fetal kernicterus would preclude pregnancy and likely maternal survival [9]. Similar concerns regarding maternal hyperbilirubinemia and risk of neonatal kernicterus have been described in the sickle cell literature. Lipsitz et al. reported a case of a pregnant sickle cell patient with a total bilirubin level of 24 mg on the day of delivery of an infant with a cord blood total bilirubin level of 10.4 mg/dL without clinical evidence of kernicterus despite presumed fetal exposure to an elevated maternal unconjugated bilirubin level for at least three weeks [8]. Our case differs from these prior reports given the long-standing in utero exposure to maternal hyperbilirubinemia (total bilirubin 1.4–7.3) for nearly three months. Yet, this chronic exposure still did not increase the risk of neonatal kernicterus. 

Similar to bilirubin, ammonia is also a neurotoxin; however, data is limited to animal literature regarding placental transfer. Through the study of pregnant mares, Johns et al. suggested that elevated maternal ammonia levels could lead to fetal encephalopathy, and pathological findings within the fetal cerebral cortex were consistent with hepatic encephalopathy despite a normal liver on necropsy [10]. Our case demonstrates the placental transfer of ammonia from the maternal compartment to the fetus given the elevated umbilical cord ammonia level at delivery. Equally important was the rapid improvement of fetal serum ammonia levels within 12 hours of life without acute neurologic sequelae signifying intact fetal hepatic function.

Although associated with adverse pregnancy outcomes, acute maternal encephalopathy due to underlying chronic liver disease does not portend adverse neonatal neurologic sequelae, notably encephalopathy, due to hyperammonemia. The care of these medically complicated women should be multidisciplinary and the potential fetal risks from routine management procedures, such as radiographically guided biliary drain exchange, must be heavily weighed against the benefit of preventing exacerbation of hepatic dysfunction. Appropriate supportive care, effective delivery, and close clinical observation can result in the successful management of this rare, yet potentially fatal, condition.

## Figures and Tables

**Table 1:** Laboratory values during hospitalization.

	On admission	Day of delivery	Day of discharge
Total bilirubin (mg/dL)	2.6	5.3	1.5
Direct bilirubin (mg/dL)	1.3	3.5	0.9
AST (units/L)	76	36	40
ALT (units/L)	27	13	14
GGT (units/L)	80	54	—
Alkaline phosphatase (units/L)	546	276	229
Amylase (units/L)	30	20	—
Lipase (units/L)	13	19	—
Ammonia (mcmol/L)	137	135	51
Electrolytes	Normal	Normal	Normal
Creatinine (mg/dL)	0.32	1.1	0.80
Albumin (g/dL)	2.6	2.2	1.4
WBC (×10^9^/L)	7.34	21.29	5.77
Hematocrit (%)	33.8	32.1	23.0
Platelets (×10^9^/L)	104	70	84
INR	1.5	1.2	1.1

—: data not available.
